# A genome-wide MeSH-based literature mining system predicts implicit gene-to-gene relationships and networks

**DOI:** 10.1186/1752-0509-7-S3-S9

**Published:** 2013-10-16

**Authors:** Zuoshuang Xiang, Tingting Qin, Zhaohui S Qin, Yongqun He

**Affiliations:** 1 Unit for Laboratory Animal Medicine, University of Michigan, Ann Arbor, MI, USA; 2 Department of Microbiology and Immunology, University of Michigan, Ann Arbor, MI, USA; 3 Center for Computational Medicine and Biology, University of Michigan, Ann Arbor, MI, USA; 4 Comprehensive Cancer Center, University of Michigan, Ann Arbor, MI, USA; 5 Bioinformatics Graduate Program, Medical University of South Carolina, Charleston, SC, USA; 6 Department of Biostatistics and Bioinformatics, Rollins School of Public Health, Emory University, Atlanta, GA 30322, USA; 7 Department of Biomedical Informatics, Emory School of Medicine, Atlanta, GA 30322, USA

## Abstract

**Background:**

The large amount of literature in the post-genomics era enables the study of gene interactions and networks using all available articles published for a specific organism. MeSH is a controlled vocabulary of medical and scientific terms that is used by biomedical scientists to manually index articles in the PubMed literature database. We hypothesized that genome-wide gene-MeSH term associations from the PubMed literature database could be used to predict implicit gene-to-gene relationships and networks. While the gene-MeSH associations have been used to detect gene-gene interactions in some studies, different methods have not been well compared, and such a strategy has not been evaluated for a genome-wide literature analysis. Genome-wide literature mining of gene-to-gene interactions allows ranking of the best gene interactions and investigation of comprehensive biological networks at a genome level.

**Results:**

The genome-wide GenoMesh literature mining algorithm was developed by sequentially generating a gene-article matrix, a normalized gene-MeSH term matrix, and a gene-gene matrix. The gene-gene matrix relies on the calculation of pairwise gene dissimilarities based on gene-MeSH relationships. An optimized dissimilarity score was identified from six well-studied functions based on a receiver operating characteristic (ROC) analysis. Based on the studies with well-studied *Escherichia coli *and less-studied *Brucella *spp., GenoMesh was found to accurately identify gene functions using weighted MeSH terms, predict gene-gene interactions not reported in the literature, and cluster all the genes studied from an organism using the MeSH-based gene-gene matrix. A web-based GenoMesh literature mining program is also available at: http://genomesh.hegroup.org. GenoMesh also predicts gene interactions and networks among genes associated with specific MeSH terms or user-selected gene lists.

**Conclusions:**

The GenoMesh algorithm and web program provide the first genome-wide, MeSH-based literature mining system that effectively predicts implicit gene-gene interaction relationships and networks in a genome-wide scope.

## Background

Biological systems are complex and involve various interactions and pathways among genes and gene products. To understand the involvement of underlying mechanism(s), exploring and defining complex relationships among genes in a genome is essential. Many types of relationships exist such as physical interactions between two proteins and regulatory interactions between multiple genes. Such gene-to-gene relationships can be found in the biomedical literature. The bibliographic database MEDLINE that can be queried through PubMed [1] contains over 20 million references of journal articles in the life sciences. Over 2,000-4,000 new entries are added daily. Each indexed article in MEDLINE is summarized in the form of manually curated Medical Subject Headings (MeSH) terms [2]. MeSH is a controlled vocabulary of medical and scientific terms for indexing articles in the PubMed literature database. The 2013 MeSH contains 26,853 MeSH descriptors organized in a hierarchal fashion based on 16 top-level categories. Over 213,000 MeSH entry terms also exist to assist in finding the most appropriate MeSH Headings [3]. All the MeSH terms are assigned to individual PubMed articles manually by knowledgeable biomedical scientists. The terminology used in MeSH provides a unique and consistent approach to retrieve information that uses different terminologies to describe similar biological and/or medical concepts.

Several approaches have been used to explore the gene-to-gene relationships and pathways reported in the literature. A common and direct strategy is to check gene co-occurrence [4,5]. Two genes may be related if they are listed in the same publication, particularly if listed in the same title, abstract, or sentence. For example, the PubGene system extracts gene relationships based on co-occurrence of gene symbols in MEDLINE titles and abstracts [5]. The PubGene co-occurrence networks display possible relationships between terms and facilitate medical literature retrieval for relevant articles implied by the network display. However, one limitation of this method is its inability to reveal direct unknown relationships among genes. Another strategy for identifying related gene pairs from the literature is to infer gene relatedness based on a common linkage to keywords. Classifications and relatedness from the co-occurrence matrix of gene names by key terms (e.g. MeSH or Gene Ontology terms) can be used to identify related gene pairs that have not been described in the title(s) or abstract(s) of any publication. This approach may be used to study co-citation and non co-citation relationships. For instance, Masys et al [6] developed a HAPI system to compare sets of genes associated with medical conditions based on the (gene names × MeSH terms) matrix. Similar methods include ARROWSMITH [7], MeSHmap [8], PubMatrix [9], and vector space modeling [10,11]. The ability to predict indirect associations among biological entities is a key feature in the linking of gene names to key terms [12,13]. However, the MeSH-based indirect approaches to infer gene-gene interactions have not been used previously for a genome-wide literature analysis. Furthermore, different methods have not been well compared. A genome-wide literature mining of gene-to-gene interactions allows ranking of the best gene interactions and investigation of comprehensive biological networks at a genome level. Advantages of a genome-wide approach in gene network analysis have been proven by numerous high throughput microarray experiments and data modeling [14].

Recently, a genome-level literature mining method has been developed by Tsoi et al. [15] to characterize human genes by Gene Ontology (GO) terms [16], *i.e*., the Ontology Fingerprint. The Ontology Fingerprint refers to a set of Gene Ontology (GO) terms that are overrepresented among the PubMed abstracts discussing a gene or biological concept together with the terms' enrichment p-value. The GO terms are employed to characterize gene functions. By comparing the Ontology Fingerprints of genes and phenotypes such as lipid levels, new relationships between genes and the phenotypes can be inferred [15].

In this study, we report a literature mining program that uses the same concepts of identifying gene relations based on gene-associated signature terms as shown in the GO-based Ontology Fingerprint study. Instead of using GO terms, we used MeSH terms to characterize genes in this report. Compared to GO terms, MeSH terms contain more comprehensive descriptions of genes including their biological and clinical knowledge. While machine-based processing is required to obtain the GO-literature association, the MeSH-literature linkages have been generated by considerably more accurate manual expert assignments. Therefore, MeSH-based literature discovery of gene-gene interactions is considered robust. In addition, our approach can be used to predict relationships between genes, which facilitate the inferring of the underlying molecular mechanisms for complex diseases. We hypothesized that MeSH could be used to predict unknown gene relationships on a genome-wide scale. Based on this hypothesis, we developed GenoMesh, a genome-wide MeSH-based literature mining algorithm that uses all literature related to a specific genome to retrieve known gene-gene associations and to infer possible novel gene-gene interactions. A web-based GenoMesh was also developed.

## Results

### The GenoMesh algorithm and functional optimization

**Figure 1:**
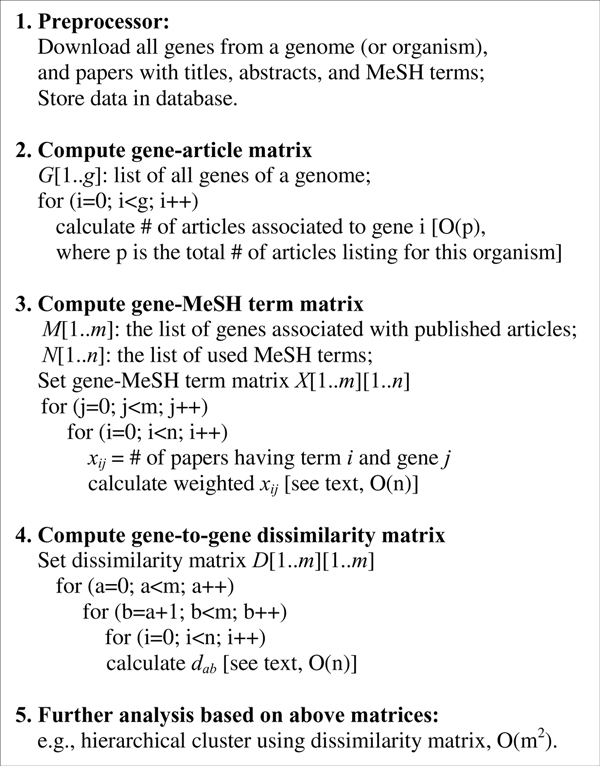
The GenoMesh algorithm.

The GenoMesh algorithm contains five steps as described in Methods and presented in Figure 1. Basically, using the titles, abstracts, and MeSH annotations of PubMed papers associated with one specific organism (*e.g*., *E. coli*), the GenoMesh algorithm calculates three matrices: gene-article matrix (Step 2 in Figure 1), gene-MeSH term matrix (Step 3), and gene-to-gene dissimilarity matrix (Step 4). The first gene-article matrix can be used for identifying the articles associating with any specific gene. Derived from the first matrix, the second gene-MeSH term matrix allows the association between MeSH terms and genes. Based on the second matrix, dissimilarity scores for any gene-gene association can be calculated. The dissimilarity scores determine how any two genes are dissociated. More details about how to implement the two organism examples (*E. coli *and *Brucella*) are described in the Methods section.

**Figure 2:**
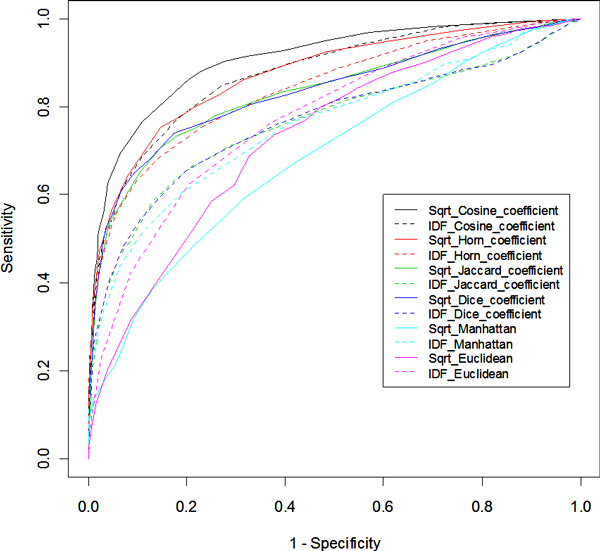
ROC curve comparison of different methods for MeSH term weighting and gene-to-gene dissimilarity calculations.

According to the gene-article matrix prepared in Step 2, a total of 1,810 *E. coli *genes were cited in at least three publications, and some 13,630 unique MeSH terms are associated with these genes. Important tasks in the GenoMesh development is the normalization of the data in the genome-wide gene-MeSH term matrix (Step 3) and optimization of the method to calculate dissimilarity scores for the construction of the gene-gene dissimilarity matrix (Step 4) (Figure 1). Each cell in the gene-MeSH matrix represents the number of articles containing a specific MeSH term related to a particular gene. Since the MeSH terms are diverse, some terms can be interpreted broadly and hence are vague, whereas others are very specific and quite informative. Consequently, rarely used words are more specific. The most frequently used weighting of MeSH terms is the TF-IDF, where TF is the term frequency, and IDF represents the Inverse Document Frequency [17]. We have tested the conventional logarithm version IDF and a newly designed variant based on a square root calculation of the IDF calculation (IDF2). After a normalized gene-MeSH matrix is generated, direct gene-to-gene relationships can be studied by preparing a gene-to-gene dissimilarity matrix. This is achieved by calculating a MeSH-based dissimilarity between any two genes (Figure 1). The dissimilarity scores between two vectors may be defined using different similarity or distance coefficient calculations [18]. The methods tested in our comparative analyses include the Jaccard index [19], the cosine coefficient [19], Dice's coefficient [19], Horn coefficient [20], and Euclidean and Manhattan distances [21]. To verify the GenoMesh algorithm and to determine which weighting scheme and similarity calculation method best fit our analysis of gene-to-gene relationships and networks, all transcription factors and their regulated genes of *E. coli *available in RegulonDB [22] were downloaded and used as the gold standard data for confirming the method. In total, 660 genes and 13,549 true relationships between these genes were used. The receiver operating characteristic (ROC) analysis was used to evaluate how well the true relationships could be predicted [23]. The overall quality of the prediction was measured by the area under the ROC curve (AUC). All 12 methods using combinations of two weighting methods and 6 dissimilarity calculation methods resulted in AUC values of 0.77-0.91. The cosine coefficient using square root weighting was proven to be the best method (AUC = 0.91) (Figure 2). These conditions were then used in all subsequent GenoMesh studies. These results also show that GenoMesh is a sensitive and specific method for calculating gene relationships.

### Weighted MeSH terms are signatures for inferring gene-gene relationships

GenoMesh annotates genes with adjusted (weighted) MeSH terms based on the associations between genes and MeSH terms as seen in the gene-MeSH matrixes. For example, *E. coli *gene *hfq *encodes for the conserved RNA-binding protein Hfq (also known as Host Factor 1). The Hfq RNA chaperone facilitates mRNA translational regulation in response to envelope stress, environmental stress and changes in metabolite concentrations [24]. *E. coli *DsrA is a small regulatory RNA that acts by RNA-RNA interactions to control translation and turnover of specific mRNAs [25]. DsrA folds into three hairpin structures. The second of these hairpin structures binds to Hfq [25]. There have been over 40 papers citing both *E. coli *Hfq and DsrA. *E. coli *protein CpxR is the congnate response regulator of the *cpxRA *two-component system that regulates biofilm formation, motility, chemotaxis, host cell invasion, and bacterial virulence [26]. The GenoMesh database contains 262 Hfq-associated articles with 500 MeSH terms, 75 DsrA-associated articles with 253 MeSH terms, and 81 CpxR-associated articles with 276 MeSH terms. Our analysis identified many shared MeSH terms associated with these three genes as illustrated in Table 1. Different MeSH terms exhibit different frequencies for each gene. The weighted MeSH term scores can be used to rank the MeSH terms. Higher-weighted MeSH terms reveal associated genes more effectively than the lower terms. It is noted that some MeSH terms (*e.g*., *E. coli*) might be too general to be very meaningful in gene function annotations. Our study does not assume these terms represent the exact functions of the genes. However, the sum of these MeSH terms is well considered as signatures for representing the knowledge about the gene. A GeneMesh program (**http://genomesh.hegroup.org/genemesh**) was developed as part of the GenoMesh web system to search all the genes associated with a particular MeSH term or all of the MeSH terms associated with a particular gene (e.g., *E. coli hfq*).

**Table 1:** Analysis of the relationships between *E. coli hfq*, *dsrA*, and *cpxR *genes

*#*	*MeSH ID*	*Term name*	*hfq papers*	*dsrA papers*	*cpxR papers*
1	D035001	host factor 1 protein	170	28	0
2	D015964	Gene Expression Regulation, Bacterial	121	36	49
3	D012333	RNA, Messenger	84	22	2
4	D022661	RNA, Untranslated	55	38	0
5	D012808	Sigma Factor	52	38	11
6	D011485	Protein binding	39	12	4
7	D014176	Protein Biosynthesis	37	16	1
8	D016601	RNA-binding Proteins	34	3	0
9	D014158	Transcription, Genetic	33	11	19
10	D001425	Bacterial Outer Membrane Proteins	31	11	20
11	D014157	Transcription Factors	24	6	13
12	D004268	DNA-Binding Proteins	22	10	3
13	D018832	Molecular Chaperones	17	3	24
14	D015536	Down-Regulation	11	0	1
15	D012270	Ribosomes	9	3	0
16	D006360	Heat-Shock Proteins	7	1	16
17	D033903	Periplasmic Proteins	1	0	13

*GenoMesh results: hfq vs dsrA: *Dissimilarity: 0.0845. p-value: 0.0003, co-published papers: 39

*hfq vs cpxR: *Dissimilarity: 0.2901. p-value: 0.0215, co-published papers: 0

The gene-MeSH matrix provides a foundation for calculation of gene-gene association. For example, the dissimilarity score between *hfq *and *dsrA *is 0.0845, and the p-value is 0.0003 (Table 1). These values indicate that *hfq *and *dsrA *are closely related. The GenePair search program (http://genomesh.hegroup.org/genepair) in GenoMesh allows looking for the gene-gene relationships for any gene pair such as *E. coli hfq*-*dsrA *pair.

### The GenoMesh algorithm predicts implicit gene relationships

Gene pair associations detected in GenoMesh can be divided into two types: 1) genes present in the same manuscript (explicit) or 2) two genes not shown in any common papers (implicit). The explicit gene relationships are usually well-studied relationships. Implicit gene-to-gene interactions with significantly low dissimilarity scores and p-values are predicted relationships since these related gene pairs are not described in the title or abstract of any given publication. As shown in Table 1, while the *E. coli hfq-cpxR *association has a p-value of 0.0215, the gene pair has not been published in even one shared paper, implying that these two genes are highly likely interacting.

To further demonstrate the utility of GenoMesh, all *E. coli*-related manuscripts were separated into two parts; articles published before 2004, and articles published afterwards. A GenoMesh analysis was performed using papers published before 2004. A number of implicit gene relationships were revealed in articles published after 2004. Selected top 5 gene pairs based on dissimilarity score ranking are listed in Table 2. All gene pairs found are critical to the same function(s) or pathway(s) indicated by the MeSH terms. For example, of the top ten gene pairs, three are interactions of three genes (*bacA*, *ybjG*, and *lpxT*) that encode three of four known undecaprenyl pyrophosphate pyrophosphatases [27]. It should be noted that gene interactions uncovered by GenoMesh contain different types of relationships and may not arise from direct physical interactions. For example, D-serine deaminase DsdA and L-serine deaminase SdaA have different and complementary roles for serine accumulation and catabolism in the colonization of the murine urinary tract by *E. coli *[28]. This does not mean, however, that they have physical interactions *in vivo*.

**Table 2:** Selected top *E. coli *five gene pairs predicated using literature data before 2004 and verified by literature data afterwards.

Index	Gene1	Gene2	Dissim Score	p-value	PMIDs	MeSH terms
1	*bacA*	*ybjG *	0.073	3.83E-05	15778224, 17660416, 18411271	Polyisoprenyl Phosphates || Bacitracin || Phosphoric Monoester Hydrolases || Fosfomycin || Periplasm

2	*nuoA *	*nuoN *	0.075	4.25E-05	15683249, 16645316, 16807239, 17489563	Electron Transport Complex I || NADH Dehydrogenase || Iron-Sulfur Proteins || NADH, NADPH Oxidoreductases || Electron Spin Resonance Spectroscopy

3	*ybjG *	*lpxT *	0.098	5.84E-05	15778224, 17660416, 18411271	Polyisoprenyl Phosphates || Bacitracin || Fosfomycin || Phosphoric Monoester Hydrolases || Periplasm

4	*hyaB *	*hybC *	0.110	7.53E-05	17668201, 17938909, 18335216	Hydrogenase || Hydrogen || Genetic Enhancement || Formate Dehydrogenases || Paraquat

5	*dsdC *	*sdaA *	0.144	1.18E-04	17785472	L-Serine Dehydratase || Serine || Amino Acid Transport Systems || Urinary Tract || Transcription Factors

### The GenoMesh algorithm effectively clusters genes on a genome-wide scale

**Figure 3:**
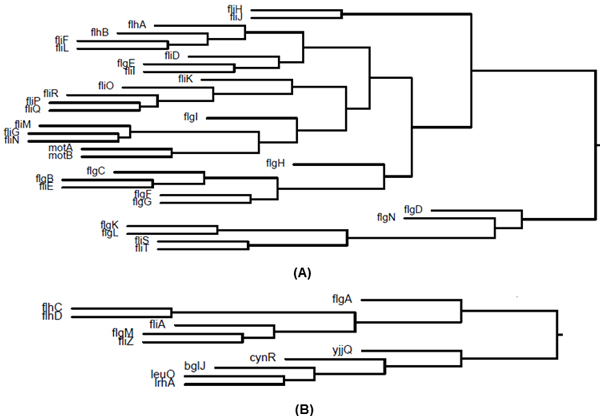
**Clusters of *E. coli *genes involved in *E. coli *flagella biogenesis**. (A) Thirty-two *E. coli *flagellar genes were clustered together; (B) Six *E. coli *flagellar genes were clustered together. The neighbour branch of the six-gene branch includes five *E. coli *genes.

**Figure 4:**
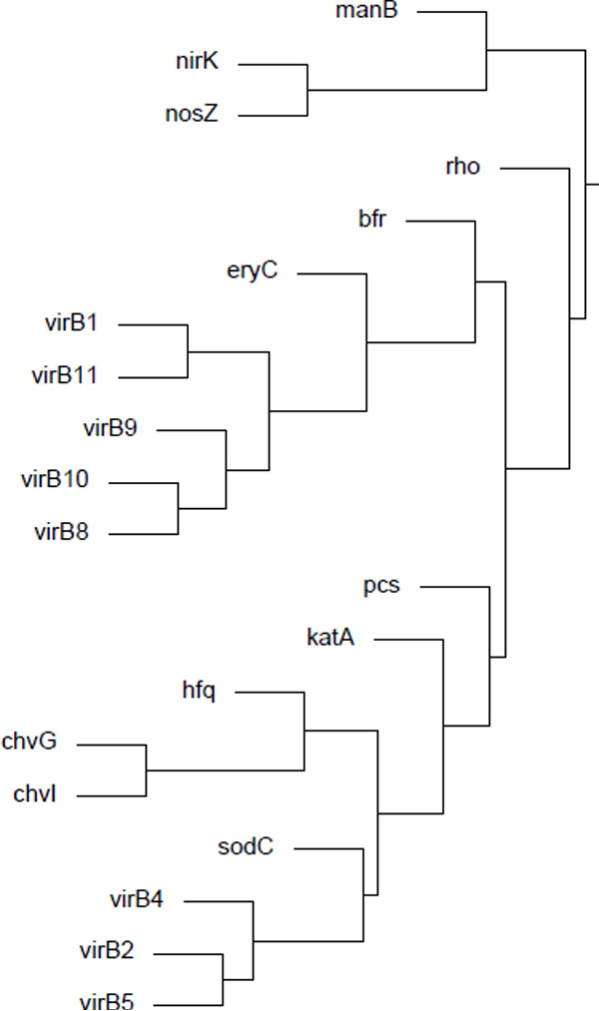
**A cluster of *Brucella *genes that includes 8 *virB *genes**.

A genome-wide gene-gene dissimilarity matrix was used to cluster all *E. coli *genes. The clustering results obtained are freely available on the GenoMesh website [29]. This cluster contains numerous gene pairs including e.g., *nfrA *and *nfrB*. Interestingly, this approach revealed information about flagella biogenesis (Figure 3). Under appropriate environmental conditions, *E. coli *synthesizes multiple flagella which facilitate motility and chemotaxis. In total 40 *fla *genes are involved in the biosynthesis of *E. coli *flagella. These occur at three loci denoted as *flg*, *flh*, or *fli*. These genes encode structural proteins, regulatory proteins, and proteins involved in assembly of flagella [30]. Two mot genes (*motA *and *motB*) are present in *E. coli*. They comprise the non-rotating components of the flagellar motor called the flagellar stator [31]. GenoMesh clusters 36 *fla *genes and the two motor genes (Figure 3A and 4B). The four flagellar genes missing in Figure 3 include *flgJ*, *fliB*, *fliC*, and *fliY*, which appeared to be associated with other *E. coli *genes. Interestingly, six *E. coli *flagellar genes were clustered close to another branch containing five other genes (*yjjQ, cynR, bglJ, leuO, lrhA*) (Figure 3B). These two sets of genes appear to share similar MeSH signatures.

The *Brucella *gene cluster is also available for download on the GenoMesh website. Compared to the large number of genes in the *E. coli *cluster, a much smaller number of *Brucella *genes are shown in the *Brucella *cluster. However, a close examination indicates that the clustering results have identified many interesting gene clusters. For example, Figure 4, a branch of the *Brucella *gene clustering hierarchy, includes several important virulence factors found in *Brucella *and suggests potential interactions among them. *Brucella *Type IV secretion, which is essential for *Brucella *pathogenesis, is encoded by the *virB *operon and includes 11 *Brucella *genes, *virB1-11*. Our analysis clusters 8 of these 11 genes. Interestingly, this cluster also includes *fliF*, an important flagellar gene [32], and *vjbR*, a quorum sensing-related transcriptional regulator [33]. It was reported that VjbR directly regulates expression of both the *virB *operon and flagellar genes either during vegetative growth or during intracellular infection [33]. The *bvrR *and *bvrS *genes encode two components (BvrS and BvrR) of a *Brucella *two-component regulatory system [34]. *Brucella hfq *encodes the RNA binding protein Hfq, which is required for the optimal stationary phase production of the periplasmic Cu, Zn superoxide dismutase SodC [35]. The BvrS/BvrR two-component regulatory system controls the internalization and early events after ingestion, whereas the intracellular trafficking beyond these early components are controlled by the VirB type IV secretion system [36]. This example demonstrates that GenoMesh can reveal hidden facts, which may lead to new insight or generate novel hypotheses.

### The GenoMesh algorithm predicts gene networks

We hypothesize that the dissimilarity values among gene pairs within any given pathway will be smaller than those from the same number of random gene pairs. The hypothesis was verified using a list of known *E. coli *pathways from the EcoCyc pathway website as the Gold Standard (Table 3). It is noted that these pathways encompass a number of different biological processes, including amino acid biosyntheses, respiration, the TCA cycle, glycolysis, fatty acid biosynthesis, and other metabolism pathways. For each pathway, the average dissimilarity score among all of the genes involved was calculated. For equal comparison, the same number of genes was randomly selected from the *E. coli *genome, and the same analysis procedure applied. The whole process was repeated 100,000 times. The Z score and empirical p-values were calculated to determine the probability of getting the same average dissimilarity score. The results obtained confirm that the GenoMesh dissimilarity measurement reveals underlying relatedness among genes in biological networks and pathways (Table 3). This study also confirms that the GenoMesh algorithm can be applied to study various biological events and pathways.

**Table 3:** GenoMesh analysis of 31 *E. coli *pathways containing at least 10 genes.

Index	Pathway name	# of genes	Average dissimilarity score	SD	Z value	*p-value
1	superpathway of chorismate	50(61)	0.077	0.134	-10.98	0

2	superpathway of histidine, purine, and pyrimidine biosynthesis	42(58)	0.080	0.117	-10.67	2.91E-275

3	superpathway of glycolysis, pyruvate dehydrogenase, TCA, and glyoxylate bypass	35(45)	0.074	0.140	-8.39	3.19E-146

4	aspartate superpathway	26(29)	0.080	0.133	-8.06	2.03E-103

5	respiration (anaerobic)	24(30)	0.086	0.170	-8.57	1.87E-108

6	respiration (anaerobic)-- electron donors reaction list	21(31)	0.209	0.260	-25.72	0

7	mixed acid fermentation	21(28)	0.102	0.171	-10.32	5.00E-138

8	superpathway of glyoxylate bypass and TCA	21(24)	0.123	0.190	-11.86	9.88E-182

9	superpathway of lysine, threonine, methionine, and S-adenosyl-L-methionine biosynthesis	21(23)	0.103	0.140	-10.45	1.71E-141

10	tRNA charging pathway	21(23)	0.073	0.107	-6.21	2.18E-51

11	superpathway of threonine metabolism	20(26)	0.133	0.208	-14.37	8.72E-253

12	superpathway of arginine and polyamine biosynthesis	19(22)	0.124	0.135	-11.32	1.46E-152

13	superpathway of phenylalanine, tyrosine, and tryptophan biosynthesis	18(25)	0.148	0.162	-15.52	1.15E-269

14	superpathway of leucine, valine, and isoleucine biosynthesis	17(30)	0.215	0.247	-23.38	0

15	aerobic respiration -- electron donors reaction list	17(21)	0.270	0.286	-30.45	0

16	TCA cycle	17(20)	0.143	0.209	-14.37	2.47E-221

17	respiration (anaerobic)-- electron acceptors reaction list	16(25)	0.194	0.212	-20.18	0

18	superpathway of lipopolysaccharide biosynthesis	15(26)	0.093	0.127	-7.47	1.20E-54

19	superpathway of glycolysis and Entner-Doudoroff	15(22)	0.114	0.126	-9.92	8.82E-95

20	superpathway of fatty acid biosynthesis	12(24)	0.223	0.221	-19.90	0

21	glycolysis I	12(18)	0.113	0.135	-8.61	1.11E-59

22	formylTHF biosynthesis I	12(15)	0.060	0.079	-3.04	4.90E-09

23	methionine and methyl-donor-molecule biosynthesis	11(13)	0.115	0.145	-8.36	1.92E-52

24	superpathway of sulfate assimilation and cysteine biosynthesis	11(12)	0.176	0.225	-14.26	2.72E-148

25	tetrahydrofolate biosynthesis I	11(12)	0.081	0.153	-4.95	1.13E-19

26	de novo biosynthesis of pyrimidine ribonucleotides	11(12)	0.119	0.142	-8.67	3.51E-56

27	peptidoglycan biosynthesis I	11(11)	0.294	0.225	-25.91	0

28	arginine biosynthesis I	11(11)	0.181	0.156	-14.93	3.35E-162

29	de novo biosynthesis of pyrimidine deoxyribonucleotides	10(18)	0.150	0.220	-11.06	4.00E-83

30	chorismate biosynthesis	10(11)	0.210	0.202	-16.58	7.45E-184

31	colanic acid building blocks biosynthesis	10(11)	0.114	0.135	-7.78	3.94E-42

Note: All permutation p-values are <0.001. * p-valule: 0 means less than 1.00E-323.

**Figure 5:**
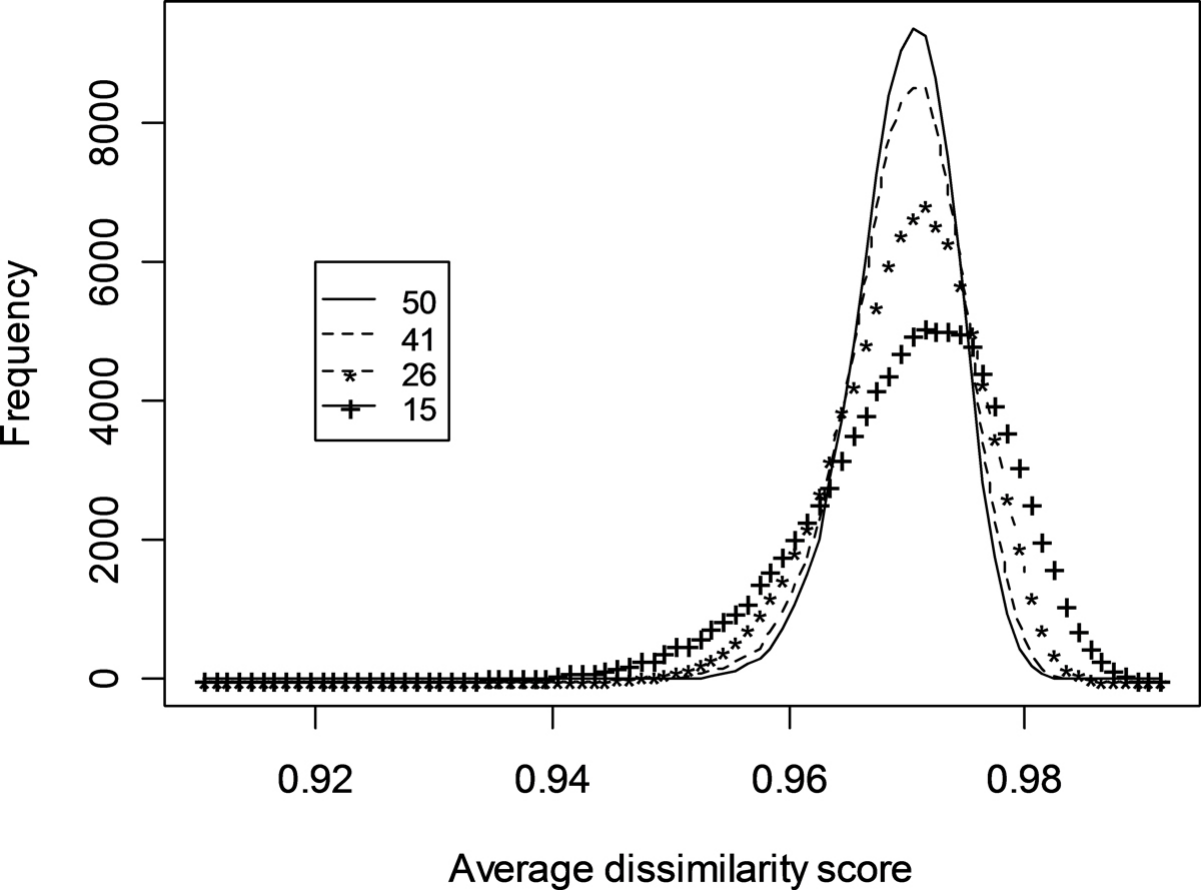
**Histogram analyses of average dissimilarity scores of random networks**. The peaks and shapes of the curves are affected by the number of genes included in the random networks.

It was also found that the distribution of the gene-gene dissimilarities from randomly selected groups of *E. coli *genes approximates the normal distribution with the peak in the range of 0.96-0.98 (Figure 5). This normal distribution profile provides a rationale and confirmation of the useful application of the GenoMesh approach to analysis of biological networks.

**Figure 6:**
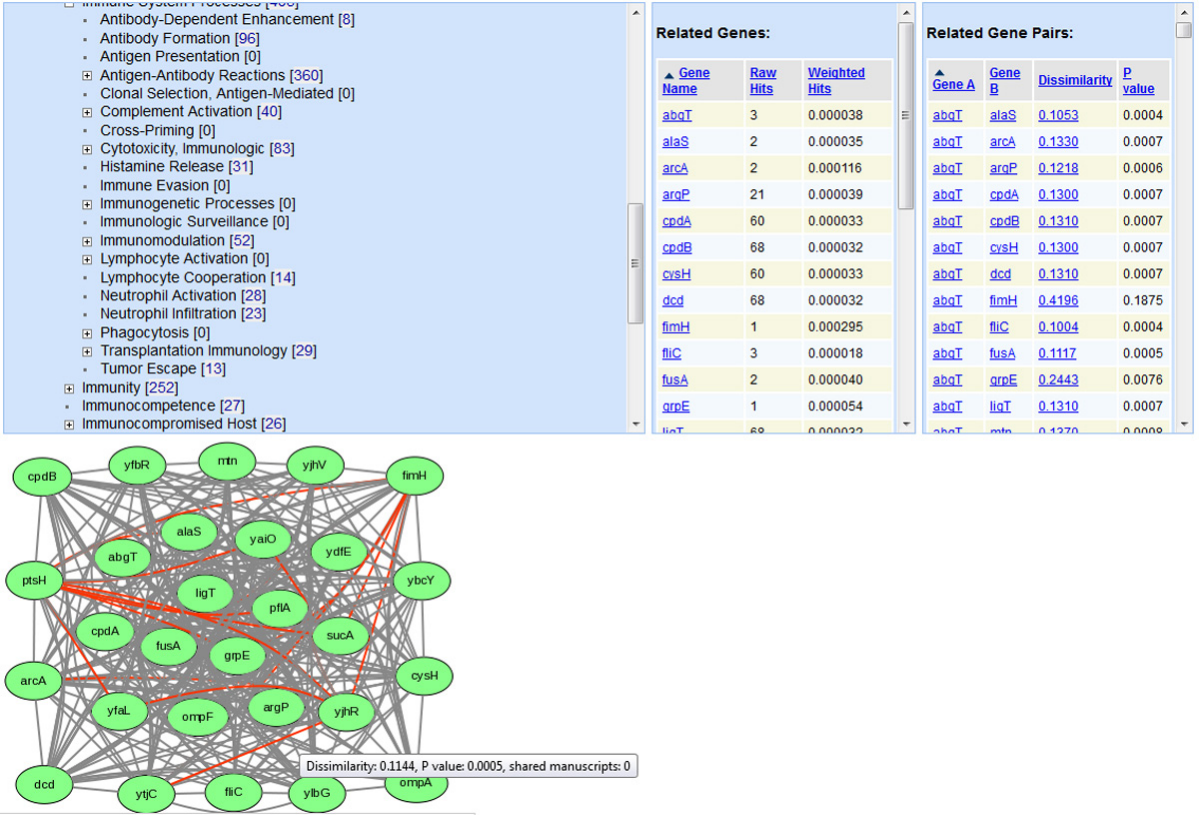
**Analysis of the term “Neutrophil Activation” from the GenoMesh MeSHBrowse website**. After browsing the MeSH hierarchical tree from “Phenomena and Processes” → “Immune System Phenomena” → “Immune System Processes” → “Neutrophil Activation”, 23 *E. coli *genes were found to be associated with the MeSH term “Neutrophil Activation". The related genes and gene pairs were then provided next to the hierarchical tree. Furthermore, a network of these 23 *E. coli *genes was automatically generated (note: the network image will only be generated if the gene number is less than 100). The gray or red-colored edges represent respectively interactions or predicted interactions. The GenoMesh annotation of the gene pair *ytjC *and *yjhR *is provided when a user moves the mouse cursor over the red line (edge) linking these two genes. A click on this link would lead the page to a detailed analysis of the gene pair (not shown).

The MeSH terms are laid out in a hierarchical tree structure. Different MeSH terms are associated with 0, 1, or many genes. Therefore, it is possible to lay out the MeSH hierarchical structure and display the genes and gene network associated with any specific MeSH term. Based on this strategy, we have developed a MeSHBrowse tool (http://genomesh.hegroup.org/meshbrowse/). For example, 23 *E. coli *genes have been found to be associated with the MeSH term “Neutrophil Activation” with a specific MeSH hierarchy (Figure 6). These 23 genes form the nodes of a gene network which includes the gene-gene associations with known literature reports (grey-colored edges) and predicted implicit gene-gene associations (red-colored edges).

To allow flexible analysis of any group of genes, a selected gene list can also be entered into to the GeneNet program (http://genomesh.hegroup.org/genenet/index.php) in the GenoMesh web system to detect the gene interaction network among the genes selected (data not shown).

### Prediction of gene relatedness by cross-species GenoMesh analysis

By comparing the GenoMesh processed results for the well-studied model organism *E. coli *and a much less-studied bacterium *Brucella *[37], it is possible to predict new gene-to-gene interactions for *Brucella *from well-studied *E. coli *gene pairs. To illustrate this, we identified a list of 5 selective genes that exist in both *E. coli *and *Brucella *and compared their associated genes in each species (Table 4). For example, *Brucella dnaK *is closely related to 12 other *Brucella *genes such as *clpP*, *dnaJ*, *groEL*, and *virB10*. Some of these genes are also found in *E. coli*. In addition, *E. coli dnaK *is closely related to over 400 other genes. It is likely that *Brucella dnaK *also has close relationship with many genes that are homologous to those *E. coli *genes. Meanwhile, some findings from *Brucella *may also help *E. coli *research. For example, *Brucella znuA *is predicted to be closely related to *purE *gene (p-value < 0.05) but not closely related to *E. coli purE *(p-value >0.05). Such a gene-gene relation in *E. coli *may deserve further investigation.

**Table 4:** Five example homologous *E. coli *and *Brucella *genes and their associated genes

Gene Name	Associated *E coli *genes	Associated *Brucella *genes
*dnaK*	abgT*, alaS, *argP, clpB, clpP, cspA, dnaJ, ftsH, grpE, groS, pflA, rcsAm uspA, ybcY, ydfE*, ... (total: 21)	*clpP, dnaJ, groEL, groES, omp25, sodC, virB10, virB11, sucB, chvL, rplL, rRNA *

*fliF*	*carB, cspA, cysH, fliC, fliE, ligT, lysR, phoQ, ompA, phoB, rpoD, rpoN, tonB, yfbY, zapA, ... (*total: 176)	*flgE, fliC, rpoD, rpoN *

*Hfq*	*bacA, csrB, deaD, deoD, dsrA, gadY, gcvB, katE, micA, oxyS, recA, rprA, rprA, rpoS, sgrS, sodC, stpA, ... (total: 118)*	*bacA, chvG, chvI, katA, sodC, virB1, virB2, virB5*

*purE*	*argD, aroE, cpdB, lysA, metE, metF, ompA, purK, pyrC, rpsE, relA, rpoB, serB*, ... (total: 519)	*chvl, omp25, omp28, sodC, virB1, virB2, wboA, znuA *

*rpoB*	*betA, dnaK, era, fliF, folD, fur, gyrA, gyrB, map, minD, polA, purE, recA, rho, secD*, ... (total: 335)	*groEL, gyrA, gyrB, katA, omp2b, parC, recA, rRNA*

*Note: To be included as an associated gene with one of the five selective target genes, the gene needs to share at least one co-publication with the target gene, or the two gene pair has a p-value < 0.05 based on the GenoMesh dissimilarity calculation.

## Discussion

In the post-genomics era, a large number of peer-reviewed articles were published at an ever increasing rate. More than 300,000 *E. coli*-related articles have been published and an additional 10,000 articles are being published each year. No single scientist or team is capable of reading all of these publications in any depth. High throughput literature mining is vital to grasp the critical information hypothesis-driven experimental design. The labor-intensive assignments of comprehensive MeSH terms in many (although not all) research areas to individual peer-reviewed papers by biomedical experts in the USA National Library of Medicine (NLM) allows the avoidance of computational annotation of PubMed papers. MeSH provides a foundation for the development of our GenoMesh text mining algorithm. MeSH contains a mixture of molecular, medical and other information that may not be appropriate to directly describe gene functions and gene relationships. However, irrelevant MeSH terms most likely will not appear in biomedical papers that study gene functions and gene relationships. At first glance, some terms (*e.g*., iron, sugar, RNA, and water) may not appear relevant or important. But, if such terms appear frequently in manuscripts describing certain genes, a possible close relationship between the gene and such terms may exist. The frequency and specificity of specific MeSH terms have also been considered in our term weighting/normalization strategy. Using MeSH terms as signatures, the genome-wide GenoMesh approach is able to predict gene relationships and pathways for various biological topics such as transcriptional factor regulations (Tables 1 and 3), flagellar biogenesis (Figure 3), neutrophil activation (Figure 6), and various other metabolic and regulatory pathways (Table 3).

GenoMesh is the first genome-wide, MeSH-based web literature mining system that annotates systematically gene functions and analyzes gene-to-gene relationships and gene networks that uses all the published manuscripts citing a single organism. The well-studied *E. coli *and less-studied *Brucella *as two distinct model organisms to GenoMesh were selected to demonstrate its feasibility. The comparative study between *E. coli *and *Brucella *also allows the generation of new insights and novel hypotheses. GenoMesh is different from many existing gene or protein interaction programs such as STRING [38] and PubGene [5] in that GenoMesh focuses on microbial gene-gene interaction identification or predictions based on genome-wide MeSH term associations and it incorporates the results from a comprehensive analysis of different dissimilarity and similarity functions.

The MeSH-based GenoMesh text mining algorithm may have some limitations. Although MeSH is designed to have a hierarchical structure outlining the relationships between different MeSH headings, the hierarchical relationships are loose and often not formally and logically defined with ontological relationship terms. A biological ontology is a set of computer- and human-interpretable terms and relations that logically represent entities in the biological world and how they relate to each other. MeSH is not considered as a formal biomedical ontology. Many ontology-based computational reasoning programs are not effectively applicable for use with the MeSH structure. MeSH, which admittedly is a very complex system, may be useful for analysis of certain biological topics but limited for study of other research topics. For example, a comparative study has shown that compared to the Vaccine Ontology (VO) [39,40], MeSH is not an ideal system to study vaccines and vaccine-related gene relationships and pathways [41]. It is possible to use VO and other biomedical ontologies to improve MeSH for better study of domain-specific gene interactions and pathways. However, the use of biomedical ontologies to replace MeSH may meet some challenges. For instance, a natural language processing (NLP)-based approach needs to be developed to assign ontology terms to individual articles. The NLP-based term assignment is very likely not as accurate as the manual annotation and MeSH term assignment to PubMed papers.

Currently the selected pair of MeSH term weighting and gene-to-gene dissimilarity is fully tested with only the *E. coli *set of documents. The reason of choosing *E. coli *is that it is a model bacterium and is associated with a large volume of publications. We have also conducted a preliminary evaluation on the pairing of MeSH term weighting and gene-to-gene dissimilarity with *Brucella *species. *Brucella *is not as well studied as *E. coli*. There is no good *Brucella *resource like *E. coli *RegulonDB that can be used to obtain gold standard data for testing our algorithm. As a result, the main criterion of our testing was based on the clustering results. The use of different selected pairs of MeSH term weighting and gene-to-gene dissimilarity resulted in different outcomes of clustering of *Brucella *genes. We found that the selected pair of MeSH term weighting and gene-to-gene dissimilarity worked for *Brucella *very well. We have demonstrated some *Brucella *gene clustering results in the manuscript.

In our study, hypothetical and unknown gene categories were excluded from our GenoMesh literature mining. These categories are the most interesting for functional inference. The initial focus in the first GenoMesh paper was to demonstrate the validity of the method. The inclusion of these categories would need to tackle a few challenges such as how to represent these genes and retrieve the information from the literature and how to evaluate the results. We plan to study these issues and possibly include such functionality in our future program development.

While the current web-based GenoMesh system provides many tools including GeneMesh, GenePair, GeneCluster, and GeneNet, these tools are under their initial stage of development and can be improved in the future. For example, the GeneMesh search program can currently search only single genes and single MeSH terms. Selection of MeSH terms requires knowing the term in advance which is not user-friendly. We plan to improve the feature by adding a possibility for users to scroll through the terms based on the structure of MeSH hierarchy. The GenePair program currently requires explicit specification of two genes. It would be better if two gene lists could be submitted. The GeneCluster is currently static and would be more useful with dynamic generation and user-friendly search capabilities. The GeneNet program can be improved with automatic prioritization and ranked result visualization. The addition of these new features would make the GenoMesh web system more useful and efficient in guiding prediction-based research.

The general GenoMesh algorithm is applicable not only to study of other microbial organisms but to study eukaryotic systems (e.g., human and mouse) and is also applicable to study the interactions between host and pathogens. One future GenoMesh research will aim to include more microbial genomes, conduct gene ortholog analysis between different microbial genomes, and evaluate the likelihood and performance of using GenoMesh to study gene-gene relations in eukaryotic systems.

## Conclusions

We have developed GenoMesh, a genome-wide, MeSH-based literature mining system that identifies direct gene-gene associations and predicts implicit interactive relationships and networks among genes within a specific genome, for example, *E. coli *and *Brucella*. The web-based GenoMesh server allows users to easily query and analyse the data generated from the GenoMesh pipeline processing. GenoMesh is a generalized literature mining program that may be applied to study gene interactions and networks in prokaryotic and eukaryotic organisms.

## Methods

### Data extraction and processing

Papers related to *E. coli *in PubMed were obtained by searching PubMed for “*E. coli*” OR “*Escherichia coli*". Papers related to *Brucella *in PubMed were obtained by searching PubMed for “*Brucella *OR brucellosis". The PubMed IDs (PMIDs), titles, abstracts, and MeSH terms of all articles related to *E. coli *and *Brucella *that had been parsed from PubMed using the PubMed literature XML format were downloaded from PubMed, including over 300,000 *E. coli*-related papers and over 15,000 *Brucella*-related papers. The parsed and downloaded literature information was then stored in a pre-defined MySQL database.

The community-based EcoGene database [16] was utilized to obtain the information of a comprehensive list of *E. coli *genes. For each gene, the information obtained from the EcoGene database includes EcoGene ID, gene symbol, gene symbol synonyms, protein name, and different protein synonyms. Normalized *Brucella *gene names were obtained from genome-wide ortholog *Brucella *gene analysis and gene name normalization as described in our previous study [41]. Basically, those ortholog genes with different names were grouped, and the different names become synonyms. A manual annotation was also applied to confirm the results of the ortholog-based grouping. In this study, each bacterial gene was identified by a primary symbol and protein name, together with a list of possible gene and protein synonyms. During text searching, gene symbols were defined as case-sensitive, except for the first letter. This approach identified and distinguished genes such as “folD” or “FolD” from the word “fold". Hypothetical and unknown genes lacking distinct gene symbols or protein names were not discussed in publications and hence discarded (Step 1 in Figure 1). For each *E. coli *or *Brucella *gene, the name matching method was used to identify all publications that contained specific gene or protein names (or their synonyms) shown in the title or abstract of each manuscript.

These publications were defined as related to the gene (Step 2 in Figure 1). From each publication identified, the MeSH terms assigned to the publication were retrieved and updated according to the MeSH term weighting as described below. From this information the gene-MeSH matrix that contains the frequency of occurrences of all MeSH terms listed for individual *E. coli *genes was formulated (Step 3). The gene-gene matrix was generated by calculating the dissimilarity score between every gene pair based on the methods described below (Step 4). Once all gene pair-wise dissimilarities were computed, all the dissimilarities were sorted, and the empirical P-value for each gene pair were calculated based on its ranked position in the sorted dissimilarity scores. Hierarchical clustering was implemented using the R hclust program (Step 5 in Figure 1).

### Optimization of weighting and dissimilarity calculations

(1) MeSH term weighting:

MeSH term weighting is based on TF*IDF [17]. Specifically, TF is the MeSH term frequency in all PubMed articles associated with a specific *E. coli *gene. IDF is the Inverse Document Frequency (IDF) used to weigh the value for each MeSH term. For a specific MeSH term *i*, IDF is first implemented using the classical logarithm method shown below:

$$\text{ID}{\text{F}}_{i}=\text{log}\left(\frac{\text{Frequency of occurence of all MeSH terms found in the literature for 33 organisms}}{\text{Frequency of occurence of MeSH term i in the E}\text{. coli literature}}\right)$$

The number of occurrences of all MeSH terms in the database is calculated by counting the total occurrences of this MeSH term in all 560,757 PubMed articles related to 33 representative bacteria or viruses as described in our publication concerned with a pathogen-host interaction data integration and analysis system (PHIDAS) [42]. Additional file 1 provides the full list of these 33 bacteria and viruses. The selection of 33 organisms other than *E. coli *alone was to make the MeSH term analysis broader in scope. The number of occurrences of the MeSH term *i *is defined as the frequency of the MeSH term in the database associated with *E. coli *only.

A separate, square root-based IDF weighting scheme was also implemented and tested:

$$\text{ID}{\text{F}}_{i}=\sqrt{\frac{\text{Frequency of occurence of all MeSH terms found in the literature for 33 organisms}}{\text{Frequency of occurence of MeSH term i in the E}\text{. coli literature}}}$$

All the terms defined in this scheme are the same as the classical logarithm method. As described in the Results section, this square root-based IDF weighting method was compared with the classical logarithm method in a Receiver Operating Characteristic (ROC) study (Figure 2).

(2) Six functions for calculating the dissimilarity score between two genes:

Six widely cited functions used for calculating distances or dissimilarity scores were explored [19-21]. The terms used are defined as follows:

*n *= number of unique MeSH terms

*X *= (*x_1_*, ..., *x_n_*), where *x_n _*= number of papers associated with term *i *for gene *a*,

*Y *= (*y_1_*, ..., *y_n_*), where *y_n _*= number of papers associated with term *i *for gene *b*,

*X *and *Y *are defined as vector representations of two genes, denoting the frequencies of MeSH terms associated with each gene. Given these definitions, the four similarity functions shown below were evaluated:

$$\text{Cosine coefficient}=\frac{\sum_{i=1}^{n}x_{i}\cdot y_{i}}{\sqrt{\sum_{i=1}^{n}x_{i}^{2}\times \sum_{i=1}^{n}y_{i}^{2}}}$$

$$\text{Jaccard coefficient}=\frac{\sum_{i=1}^{n}x_{i}\cdot y_{i}}{\sum_{i=1}^{n}x_{i}+\sum_{i=1}^{n}y_{i}-\sum_{i=1}^{n}x_{i}\cdot y_{i}}$$

$$\text{Dice coefficient}=\frac{2\sum_{i=1}^{n}x_{i}\cdot y_{i}}{\sum_{i=1}^{n}x_{i}+\sum_{i=1}^{n}y_{i}}$$

$$\text{Horn coefficient}=\frac{2\sum_{i=1}^{n}x_{i}\cdot y_{i}}{\sum_{i=1}^{n}x_{i}^{2}+\sum_{i=1}^{n}y_{i}^{2}}$$

Two dissimilarity functions were also implemented:

$$\text{Manhattan distance }=\sum_{i=1}^{n}|x_{i}-y_{i}|$$

$$\text{Euclidean distance }=\sqrt{\sum_{i=1}^{n}{\left(x_{i}-y_{i}\right)}^{2}}$$

(3) Calculation of dissimilarity scores based on weighted MeSH terms:

The revised dissimilarity measure (*D_M_*) based on the Cosine coefficient is defined as:

$$D_{M}=1-\frac{\sum_{i=1}^{n}w_{i}^{2}\cdot x_{i}\cdot y_{i}}{\sqrt{\sum_{i=1}^{n}w_{i}^{2}\cdot x_{i}^{2}\times \sum_{i=1}^{n}w_{i}^{2}\cdot y_{i}^{2}}}$$

where *i *is a specific MeSH term, *w_i _*is the weight assigned to the *i*th MeSH term (one of the two IDF-based weighting schemes). In the Cosine coefficient formula, the *x_i _*and *y_i _*have been changed to (*w_i;_x_i_*) and (*w_i;_y_i_*), respectively. The dissimilarity scores based on other similarity coefficients are defined in a similar manner.

The revised dissimilarity measure (*D_M_*) based on the Manhattan distance is defined as:

$$D_{M}=\sum_{i=1}^{n}|w_{i}x_{i}-w_{i}y_{i}|$$

where the variables are defined as the same as shown above. The revised dissimilarity measure based on the Euclidean distance is defined similarly.

(4) Verification and optimization of MeSH term weighting and dissimilarity score calculation

To test whether the actual quantitative value in the MeSH term dissimilarity measure is indicative of the relationships of the two selected genes, the ROC analysis was applied [23]. Genes from 13,549 gene pairs of transcriptional factors and their individually regulated genes available in RegulonDB [22] were used as the gold standard. The calculation methods described above were used to calculate the specificity and sensitivity of analyzing the gene-gene relationships using the true gene pairs in the gold standard data compared to the same number of randomized gene pairs in the GenoMesh database. One hundred gene pairs were selected randomly from the standard set, and 100 pairs were selected randomly from the GenoMesh database. The true positive rate (Sensitivity) and false positive rate (1-Specificity) were then calculated based on gradually increasing dissimilarity cut-off values (between 0 and 1). The calculations were repeated 100 times and the averages recorded. A ROC curve was plotted for all sets of data to verify the GenoMesh algorithm and to optimize the method of calculating a MeSH-based dissimilarity score based on data in the literature.

### Development of the GenoMesh web server

The GenoMesh web server (http://genomesh.hegroup.org) was developed using a three-tier architecture built on two HP ProLiant DL380 G6 servers which run the Redhat Linux operating system (Redhat Enterprise Linux ES 5). Users can submit database or analysis queries through the web. The queries are processed using PHP/Perl/SQL (middle-tier, application server based on Apache) against a MySQL (version 5.0) relational database (back-end, database server). The result of each query is presented to the user in the web browser. Two servers are regularly scheduled to backup each other's data. The GenoMesh system currently contains five programs: 1) GeneMesh, searching MeSH terms (or genes) from a gene (or MeSH) query; 2) GenePair, analysing a designated gene pair; 3) GeneCluster, displaying the hierarchical clustering results; 4) GeneNet, predicting a gene interaction network based on a user-defined gene list; 5) MeSHBrowse, browsing MeSH tree for MeSH terms and predicted genes and gene interaction network for each MeSH term. General MeSH terms and structures are extracted from the MeSH website (http://www.nlm.nih.gov/mesh). The images of the interaction networks are generated automatically with an internally developed script based on the graph visualization software Graphviz (http://www.graphviz.org).

### Prediction of gene-to-gene relationships and networks using GenoMesh

To test the ability of GenoMesh to predict gene-to-gene interactions lacking direct literature support, all *E. coli *literature data were separated into two parts, literature published before January 1, 2004 and after January 1, 2004. The literature published before 2004 was used for predicting gene-to-gene interactions. The results were verified using the results published after 2004.

To evaluate whether gene pairs in the same pathway have lower GenoMesh dissimilarity scores than gene pairs from a random group of genes, a list of known *E. coli *pathways from the EcoCyc pathway website [43] was collected. To avoid uncertainties attributed to minor pathways, pathways containing less than ten genes were excluded. For biological pathways containing *N *related genes, the GenoMesh dissimilarity value for all *n*(*n*-1)/2 gene pairs *d_ij_, i, j *= 1, ..., *n*, was calculated, and the average

$${\bar{d}}^{*}=\frac{\sum_{1\leq i<j\leq n}d_{ij}}{n\left(n-1\right)/2}$$

taken as the average GenoMesh value for the pathway. *N *genes were randomly selected from the *E. coli *genome and their pair-wise dissimilarity values calculated. The average of these values is denoted as *d_0_*. The same procedure was repeated 100,000 times to obtain *d_i,_^0 ^i *= 1, ..., 100,000. The value obtained was used to approximate the null distribution of the average GenoMesh value for gene groups of size *N*. The empirical p-value was calculated as

$$p_{e}=\frac{\sum_{i=1}^{N}I_{\left[\bar{d}{}^{*}\leq {\bar{d}}_{i}^{0}\right]}}{N}.$$

The sample mean

$$\mu_{0}=\frac{\sum_{1\leq i<j\leq s}d_{ij}}{S\left(S-1\right)/2}$$

and variance

$$\sigma_{0}^{2}=\frac{\sum_{1\leq i<j\leq s}d_{ij}^{2}}{S\left(S-1\right)/2}-\mu_{0}^{2}$$

of the sample of *all *pair-wise GenoMesh values can be estimated. Basically, such a p-value is a permutation p-value determined empirically by repeating the same process many times to see how many times the test result was significant. There is only one test. Therefore, a multiple test correction is not required.

For pathways with large *n*(*n*-1)/2 values, the central limit theorem can be used to derive the asymptotic distribution for average GenoMesh values for a random group of *n *genes, which is normal with mean *μ *and variance 2*σ_0_^2^*/(*n*(*n*-1)). Hence the asymptotic z-value can be calculated as

$$Z=\frac{\bar{d}{}^{0}-\mu_{0}}{2\sigma_{0}^{2}/\left(n\left(n-1\right)\right)}$$

The exhaustive MeSH term dissimilarity value calculations for all of the possible *E. coli *gene pairs allows analysis of the relatedness of gene pairs without using reported studies (no overlapped references).

## List of abbreviations used

MeSH: Medical Subject Headings; NCBI: National Center for Biotechnology Information; IDF: Inverted Document Frequency; TF: Term Frequency; ROC: Receiver Operating Characteristic; AUC: (ROC) Area Under Curve; GO: Gene Ontology; VO: Vaccine Ontology; XML: Extensible Markup Language.

## Competing interests

The authors declare that they have no competing interests.

## Authors' contributions

ZX implemented the GenoMesh algorithm and developed the GenoMesh web program as the primary software developer and database administrator. TQ supported data analysis. ZSQ provided statistics expertise and participated in project design and result interpretation. YH co-designed the project, performed data analysis, served as a secondary software developer and database administrator, and wrote the draft. All authors participated in manuscript editing and discussions.

## Supplementary Material

Additional File 1 **Supplemental Table 1**. Thirty three pathogens used to calculate the MeSH term frequencies. Click here for file

## Declaration

The funding for the paper publication was supported by NIH-NIAID grant 1R01AI081062 to YH.

This article has been published as part of *BMC Systems Biology *Volume 7 Supplement 3, 2013: Twelfth International Conference on Bioinformatics (InCoB2013): Systems Biology. The full contents of the supplement are available online at http://www.biomedcentral.com/bmcsystbiol/supplements/7/S3.
