# Supplementary material for: A genome-wide MeSH-based literature mining system predicts implicit gene-to-gene relationships and networks
Source: BMC Syst Biol. 2013 Oct 16;7(Suppl 3):S9. doi: 10.1186/1752-0509-7-S3-S9 (PMC3852244; doi:10.1186/1752-0509-7-S3-S9)
Supplement: Additional File 1 — Supplemental Table 1. Thirty three pathogens used to calculate the MeSH term frequencies. [file 1752-0509-7-S3-S9-S1.doc]

**Additional File:**

**Supplemental Table 1.** Thirty three pathogens used to calculate the MeSH term frequencies.

| index | Pathogen name |
| --- | --- |
| 1 | *Bacillus anthracis* |
| 2 | *Brucella* |
| 3 | *Burkholderia* |
| 4 | *Campylobacter jejuni* |
| 5 | *Clostridium perfringens* |
| 6 | *Coxiella burnetii* |
| 7 | *Escherichia coli* |
| 8 | *Francisella tularensis* |
| 9 | *Helicobacter* |
| 10 | *Legionella pneumophila* |
| 11 | *Listeria monocytogenes* |
| 12 | *Mycobacterium tuberculosis* |
| 13 | *Rickettsia prowazekii* |
| 14 | *Salmonella* |
| 15 | *Shigella* |
| 16 | *Vibrio* |
| 17 | *Yersinia pestis* |
| 18 | Crimean-Congo hemorrhagic fever virus |
| 19 | Foot-and-mouth disease virus |
| 20 | Guanarito virus |
| 21 | Human immunodeficiency virus |
| 22 | Junin virus |
| 23 | Lake Victoria marburgvirus |
| 24 | Lassa virus |
| 25 | Louping ill virus |
| 26 | Machupo virus |
| 27 | Measles virus |
| 28 | Reston ebolavirus |
| 29 | Rift Valley fever virus |
| 30 | Vaccinia virus |
| 31 | Variola virus |
| 32 | Venezuelan equine encephalitis virus |
| 33 | Yellow fever virus |
